# Reliable wolf-dog hybrid detection in Europe using a reduced SNP panel developed for non-invasively collected samples

**DOI:** 10.1186/s12864-021-07761-5

**Published:** 2021-06-25

**Authors:** Jenni Harmoinen, Alina von Thaden, Jouni Aspi, Laura Kvist, Berardino Cocchiararo, Anne Jarausch, Andrea Gazzola, Teodora Sin, Hannes Lohi, Marjo K. Hytönen, Ilpo Kojola, Astrid Vik Stronen, Romolo Caniglia, Federica Mattucci, Marco Galaverni, Raquel Godinho, Aritz Ruiz-González, Ettore Randi, Violeta Muñoz-Fuentes, Carsten Nowak

**Affiliations:** 1 Ecology and Genetics Research Unit, University of Oulu, Oulu, Finland; 2 Conservation Genetics Group, Senckenberg Research Institute and Natural History Museum Frankfurt, Gelnhausen, Germany; 3 Institute for Ecology, Evolution and Diversity, Johann Wolfgang Goethe-University, Biologicum, Frankfurt am Main, Germany; 4 LOEWE Centre for Translational Biodiversity Genomics (LOEWE-TBG), Frankfurt am Main, Germany; 5 Association for the Conservation of Biological Diversity, Focşani, Romania; 6 Department of Systems Ecology and Sustainability, Faculty of Biology, University of Bucharest, Bucharest, Romania; 7 Department of Veterinary Biosciences, University of Helsinki, Helsinki, Finland; 8 Department of Medical and Clinical Genetics, University of Helsinki, Helsinki, Finland; 9 Folkhälsan Research Center, Helsinki, Finland; 10 Natural Resources Institute Finland (Luke), Eteläranta 55, FI-96300 Rovaniemi, Finland; 11 Department of Biology, Biotechnical Faculty, University of Ljubljana, Ljubljana, Slovenia; 12 Department of Biotechnology and Life Sciences, Insubria University, Varese, Italy; 13 Unit for Conservation Genetics (BIO-CGE), Department for the Monitoring and Protection of the Environment and for Biodiversity Conservation, Italian Institute for Environmental Protection and Research, Bologna, Italy; 14 Scientific Area, WWF Italy, Rome, Italy; 15 CIBIO/InBIO, Centro de Investigação em Biodiversidade e Recursos Genéticos, Universidade do Porto, Campus de Vairão, 4485-661 Vairão, Portugal; 16 Department of Biology, Faculty of Science, University of Porto, Porto, Portugal; 17 Department of Zoology and Animal Cell Biology, Zoology Laboratory, University of the Basque Country (UPV/EHU), Vitoria-Gasteiz, Spain; 18 Department of Biological, Geological and Environmental Sciences, University of Bologna, Bologna, Italy; 19 Department of Chemistry and Bioscience, Faculty of Engineering and Science, University of Aalborg, Aalborg, Denmark; 20 ​European Molecular Biology Laboratory, European Bioinformatics Institute, Wellcome Trust Genome Campus, Hinxton, Cambridge, UK

**Keywords:** *Canis lupus*, *Canis lupus familiaris*, Hybridization, SNP genotyping, Non-invasive sampling, Museum samples

## Abstract

**Background:**

Understanding the processes that lead to hybridization of wolves and dogs is of scientific and management importance, particularly over large geographical scales, as wolves can disperse great distances. However, a method to efficiently detect hybrids in routine wolf monitoring is lacking. Microsatellites offer only limited resolution due to the low number of markers showing distinctive allele frequencies between wolves and dogs. Moreover, calibration across laboratories is time-consuming and costly. In this study, we selected a panel of 96 ancestry informative markers for wolves and dogs, derived from the Illumina CanineHD Whole-Genome BeadChip (174 K). We designed very short amplicons for genotyping on a microfluidic array, thus making the method suitable also for non-invasively collected samples.

**Results:**

Genotypes based on 93 SNPs from wolves sampled throughout Europe, purebred and non-pedigree dogs, and suspected hybrids showed that the new panel accurately identifies parental individuals, first-generation hybrids and first-generation backcrosses to wolves, while second- and third-generation backcrosses to wolves were identified as advanced hybrids in almost all cases. Our results support the hybrid identity of suspect individuals and the non-hybrid status of individuals regarded as wolves. We also show the adequacy of these markers to assess hybridization at a European-wide scale and the importance of including samples from reference populations.

**Conclusions:**

We showed that the proposed SNP panel is an efficient tool for detecting hybrids up to the third-generation backcrosses to wolves across Europe. Notably, the proposed genotyping method is suitable for a variety of samples, including non-invasive and museum samples, making this panel useful for wolf-dog hybrid assessments and wolf monitoring at both continental and different temporal scales.

**Supplementary Information:**

The online version contains supplementary material available at 10.1186/s12864-021-07761-5.

## Background

Gray wolves (*Canis lupus*) are currently expanding to areas in Europe from which they had been temporarily absent [1]. This increase in population size and range is due to effective legal protection measures, reforestation, expansion of wild ungulate populations, and increased public awareness. During the last three decades, wolves have increased in numbers in several regions in Europe, including Fennoscandia (e.g. Finland, Sweden), the Alps (e.g. France, Italy, Switzerland), Central Europe (e.g. Czech Republic, Germany, western Poland) and the northern part of the Iberian Peninsula [2, 3]. In many of these regions, a wealth of genetic data on wolf dispersal has been collected over the years to track the recolonization process (e.g. [4–9]).

Analyses based on genetic markers, such as microsatellites and mitochondrial sequences, have greatly improved our knowledge of wolves, including estimates of pack structure, population censuses and effective population sizes, and inference of the population of origin for migrating individuals, among others (see [2]). Further, microsatellites markers, either solely or in combination with other markers, have been used to assess the admixture of wolves and domestic dogs (*C. l. familiaris*); reported rates of admixed animals in local wolf populations range between 0 and 10% (e.g., [10–14], but see for instance [15] for locally higher admixture rates). However, identification of wolf-dog hybrids based on microsatellite data is far from trivial, due to the low number of alleles with distinctive frequencies between wolves and dogs, the rather limited number of loci used in many studies, and the fact that results strongly depend on reference samples and the extent of population substructure in the dataset [14, 16–18]. Moreover, the fact that most laboratories have relied on different panels of microsatellite markers has hampered the comparability of data on wolf-dog admixture across populations, limiting our knowledge on the extent of hybridization [2].

Genome-wide approaches have allowed previously unattainable resolution in wolf-dog hybrid identification, such as later-generation hybrids and the differentiation of ancient and recent hybridization events [19–21]. Analyses have confirmed the genetic separation of wolves and dogs, but also found strong support for widespread existence of historic introgression of dog DNA in virtually all wolf populations across Europe, Asia and North America [20, 22, 23]. These results have unveiled a complex evolutionary history of wolves, in which hybridization occurring at multiple time scales nevertheless resulted in wolves maintaining their genetic distinctiveness from dogs. While such genome-wide approaches importantly contributed to our knowledge on wolves, their application in routine wolf monitoring for wildlife management purposes is unpractical due to high costs, extensive analysis procedures and the requirement for high-quality DNA samples [24]. Genetic wolf monitoring, however, often relies on the analysis of numerous non-invasively collected samples, in which DNA is often in low-quantity and has low-quality [25].

Here we describe the development of a Single Nucleotide Polymorphism (SNP) panel selected for maximum discrimination power between European wolves and dogs that allows for the reliable identification of pure and admixed individuals. The method relies on the utilization of a microfluidic array designed to simultaneously genotype 96 SNPs from 96 samples, which we have optimized for samples with low DNA quality and quantity. The method works reliably with all sample types commonly collected in wolf monitoring, including scats or saliva traces from wolf kills and, notably, also museum samples. The results are readily comparable across different laboratories, making this method suitable to comprehensively assess hybridization of wolves and dogs at both local and continental scales.

## Results

### Assay performance

Genotyping success with the selected panel was high across samples and markers. Only 2.7% of the samples failed in all reactions (*n* = 14) and hence were discarded from further analyses. The average genotyping success rate was 0.97. As expected, genotyping success was the highest for concentrated buccal swabs (1.00) and tissue samples (0.99), while it was only slightly lower (0.93–0.97) for the other sample types, including museum samples (Table 1).

**Table 1:** Genotyping success rates (proportion of successfully scored loci over the 93 genotyped SNP loci) for different samples types. “Removed samples” were not included in the calculations due to genotyping failure for all markers

Sample type	Samples (*n*)	Removed samples (*n*)	Genotyping success rate (%)
Tissue	149	1	99
Concentrated buccal swab	28	0	100
Saliva swab	13	1	97
Hair	10	0	95
Scat	63	2	93
Urine	4	0	97
Blood	3	0	96
Museum samples	40	6	97

Genotyping consistency was also generally high. When genotypes of high-quality samples (tissue) were compared with non-invasively collected samples from the same individual, we detected only one individual with one allele in the non-invasive sample that was not found in the tissue sample (0.04% rate), as well as one missing allele in three different non-invasive samples (2.88% rate), while the missing data was obtained for 0–12 loci per sample (2.42% rate) (Table S1). When comparing results from 22 tissue samples with the Illumina CanineHD chip results and assuming that the Illumina genotype was the correct one, only one allelic discrepancy was found, namely a missing allele in the Fluidigm genotype (0.49% rate) (Table S2).

Cross-species amplification testing resulted in valid genotypes only for other Canidae species (Table S3). Samples from golden jackals produced genotypes with 0.97–0.99 genotyping success rates and red foxes (three out of four) genotypes with 0.77, 0.78 and 0.85 call rates. No successful amplifications were observed for the case of the tested prey species for wolves (roe and red deer, wild boar, goat, and sheep), nor for humans or carnivores that are not members of Canidae.

### Allele frequencies in wolves and dogs using the selected SNP panel

**Fig. 1:**
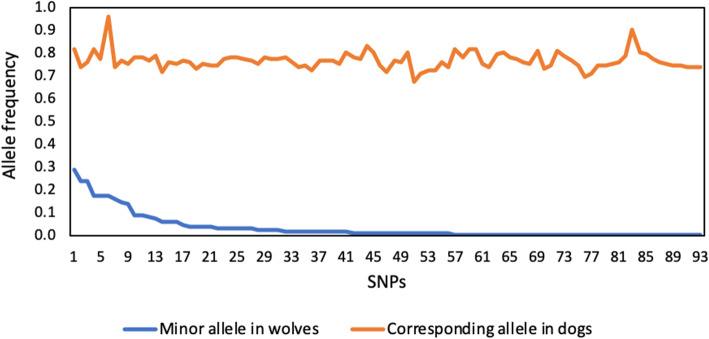
Allele frequencies for the 93 selected SNPs in wolves and dogs. High discriminating power is due diverging allele frequencies in the wolf and dog groups, accompanied by the presence of private alleles for dogs

*F*_ST_ calculated for each of the 93 SNPs in our panel indicated high discriminatory power between wolves and dogs (*F*_ST_ = 0.40–0.88; average 0.70). All markers were polymorphic in dogs, with allele frequencies > 0.10, except for one (BICF2P263751, allele frequency = 0.04), and most markers had one allele with frequency 0.7–0.8. Wolves, on the contrary, had 18 markers with a fixed allele (all populations considered) and 77 markers had one allele that was rare (frequency < 0.1). For all markers, the most frequent allele in one species was the least frequent in the other (or absent, in the case of wolves; Fig. 1).

### Population differentiation and admixture analysis

Using this final SNP panel, wolves (*n* = 288) and dogs (*n* = 300; excluding wolf-dog breeds, *n* = 14) were significantly differentiated (*F*_ST_ = 0.72, *p* < 0.05), and so were wolf-dog breeds (*n* = 14) and dogs (*n* = 300; *F*_ST_ = 0.20, *p* < 0.05). Different wolf populations also showed significant differentiation. The divergence was highest between wolves from Italy and other European populations, with *F*_ST_ = 0.17–0.28 (*p* < 0.05; Table S4). Wolves from the Iberian Peninsula showed lower divergence from wolves from Central and Eastern Europe (*F*_ST_ = 0.07–0.17, *p* < 0.05) and there was very low divergence between remaining wolf populations (*F*_ST_ = 0.03–0.11).

**Fig. 2:**
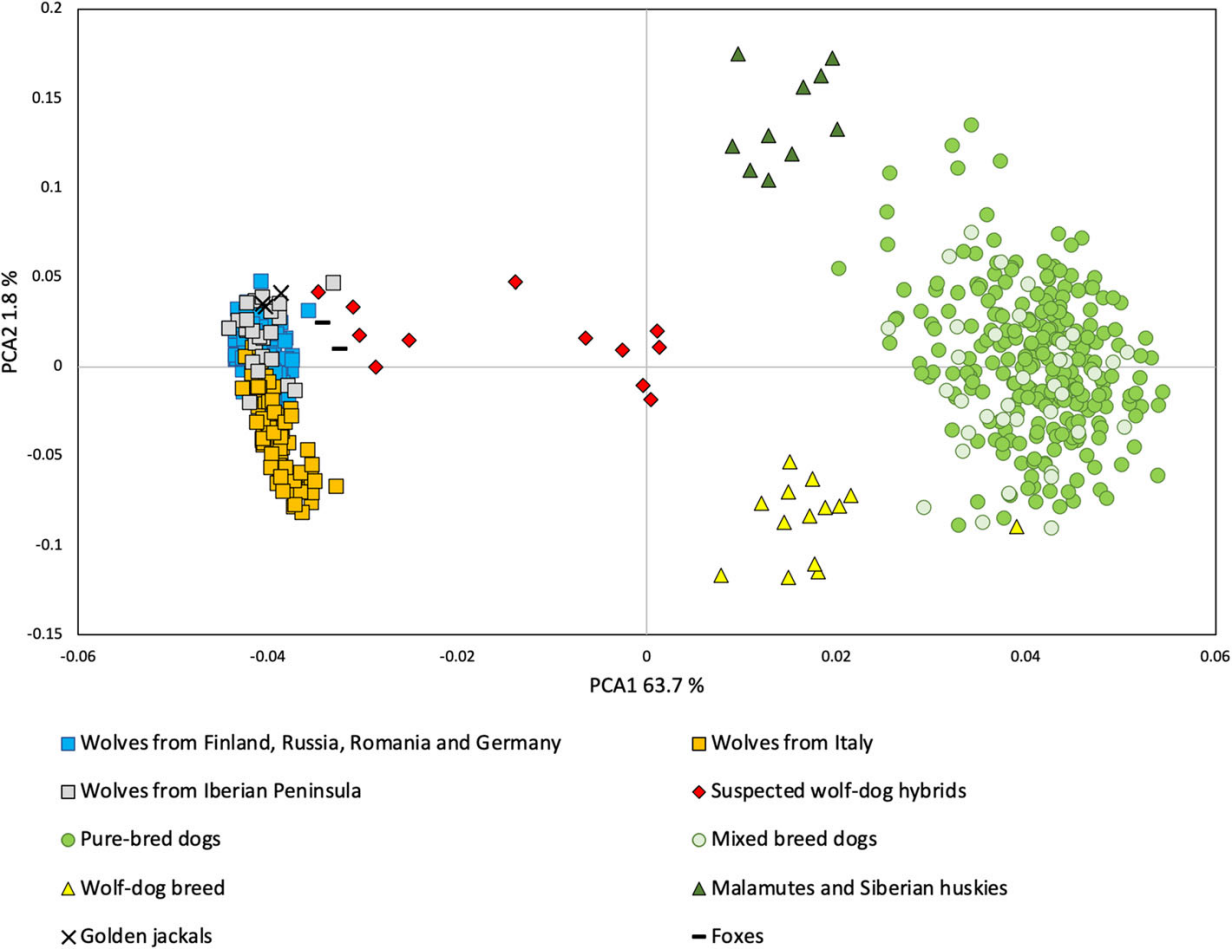
Principal component analysis (PCA) based on 93 SNPs selected to maximize discriminatory power between wolves and dogs. Wolves are color-coded based on sampling locations, except seven immigrant wolves from the Alpine population sampled in Germany that were color-coded as wolves from Italy (in agreement with previous microsatellite and haplotype data, see text). Purebred dogs were sampled in Finland and non-pedigree dogs in Germany and Romania. Saarloos Wolfdogs and Czechoslovakian Wolfdogs were sampled in Finland and Germany. Suspected wolf-dog hybrids were identified based on previous microsatellite analysis and ancillary evidence (see text). Foxes and golden jackals were included to assess cross-species amplification

A PCA analysis (Fig. 2) of multilocus genotypes based on the selected 93 markers reflected substantial differentiation between wolves and dogs and showed more genetic diversity in dogs than in wolves. Individuals identified as suspected hybrids were either placed in an approximately equidistant position between the wolf and dog clusters or closer to the wolf cluster. Wolves formed one tight cluster, including both contemporary and museum samples, as well as those from the animal parks. Wolves sampled in Italy and the seven immigrants from the Alpine population that were sampled across Germany clustered together and only partially overlapped with the remaining wolves (shown more clearly in Figure S1, PCA for only wolves). Golden jackals clustered closely with wolves, while foxes were slightly separated. The PC1-axis discriminated wild canids from dogs, while the PC2-axis explained some of the variation found in wolves and dogs. Wolf-dog breeds were located close to the dog cluster, but were closer to the wolf cluster than other dog breeds. A similar pattern was observed for Siberian Huskies and Alaskan Malamutes, with the PC2-axis separating the artic breeds from the wolf-dog breeds.

Clustering analysis implemented in STRUCTURE [26] assigned wolves and dogs to two distinct clusters. When all the wolves and dogs were analyzed together, wolves had individual assignment values *q*_w_ > 0.93 and except for two individuals from Germany (one an immigrant from the Alpine population), one from Italy and three from the Iberian Peninsula, all assignment values were *q*_w_ > 0.97 (Table S5). Suspected hybrids had assignment values (*q*_w_ = 0.52–0.92; Table 2), in agreement with previous knowledge (Table S6). Dogs were preferentially assigned to the other cluster, showing higher variation in assignment values (q_d_ = 0.59–1.00). Out of the dog breeds, Siberian Huskies and Alaskan Malamutes had the lowest assignment values (q_d_ = 0.59–0.73). Similarly, other dog breeds with roots in Siberia (East Siberian Laika, West Siberian Laika, Russo-European Laika and Samoyed) had somewhat lower assignment values (on average q_d_ = 0.86). Individuals from the wolf-dog breeds (Saarloos Wolfdog and Czechoslovakian Wolfdog) had a wider range of assignment values (q_d_ = 0.64–0.99), with an average assignment value q_d_ = 0.73. The remaining purebred dogs and non-pedigree dogs had assignment values q_d_ = 0.84–1.00.

**Table 2:** Results from NEWHYBRIDS and STRUCTURE analyses for suspected hybrids of wolves and dogs. Analyses were run with the four possible prior combinations (see main text). The range of results from different runs is indicated. Assignment values based on STRUCTURE *q*_w_ values were obtained for *K* = 2

		NEWHYBRIDS		STRUCTURE
Origin	ID	Assigned Category	*q*_i_	Wolf *q*_w_
Germany	GW01xf	F1	1.00	0.56
	GW02xm	F1	1.00	0.54
	GW03xm	F1	1.00	0.54
Romania	RO022m	BC2w	0.98–0.99	0.85
Czech Republic	GW05xf	F1	1.00	0.52
Finland	CL134	F1	1.00	0.55
	CL370	F1	1.00	0.52
	CL309	F2	1.00	0.63
	CL307	BC1w	0.81–0.98	0.76
	CL308	BC2w	0.98–0.99	0.83
	CL419	BC2w	0.74–0.92	0.89
	CL420	BC2w/BC3w	0.59–0.64/0.81–0.84	0.92

When performing the analysis for wolf samples only, the most likely number of populations was *K* = 2 (Figure S2), which separated wolves from Italy from the remaining wolves (Figure S3). A less supported *K* = 3 assigned Iberian wolf samples to another cluster (Figure S4). Because differentiation between populations was significant, we also performed separate runs with *K* = 2 for wolves from Central and Eastern Europe (including Finland, Russia, Romania and Germany), Italy and the Iberian Peninsula, with dogs. These analyses assigned all the wolves to one cluster *q*_w_ > 0.97 (Table S5). Despite somewhat higher assignment values of some of the wolves to that cluster, the assignments were similar than in the run including all the wolf samples.

NEWHYBRIDS [27] analyses were run four times (with four different prior combinations based on the two available priors, Jeffreys and Uniform) for all individuals together, without prior assumption of parental populations. All assumed wolf individuals, including museum samples from Finland and wolves from animal parks (Table S7; Table S8), were categorized as wolves (*q*_i_ > 0.87 using Uniform priors and *q*_i_ > 0.93 when using Jeffreys priors for theta, *q*_i_ = 1 for 283–284 individuals depending on the priors). The 12 suspected wolf-dog hybrids were assigned to different hybrid categories in NEWHYBRIDS (Table 2), in agreement with our field observations (Table S6). As for the dogs, most of the purebred individuals, except for wolfdogs, were classified as dogs (224–228 out of 264 individuals, depending on the priors used). The individuals that were not classified as dogs but rather as hybrids (F2, BC1d or BC2d) were mostly from breeds with Siberian roots (*n* = 27–28) or wolf-dog breeds (*n* = 13), which also had the lowest STRUCTURE assignment values among dogs. Among non-pedigree dogs, 29–30 out of 36 were assigned as dogs, while the rest were assigned as BC2d or were not clearly assigned to any category (posterior probability < 0.5 to several categories). All the samples from golden jackals and red foxes were categorized as wolves in NEWHYBRIDS and assigned to the wolf cluster in STRUCTURE, with assignments to wolves *q*_w_ = 0.95–0.98 (Table S9).

For testing purposes, and because there was significant pairwise genetic differentiation between different populations, we also performed three separate runs for different sample sets (wolves from Central and Eastern Europe and dogs, wolves from the Alpine population and dogs, and wolves from the Iberian Peninsula and dogs). The categorizations of individuals were very similar, but the assignment values of wolves to wolf cluster were higher (Table S5), as expected when dataset is more homogenous. Hybrids were assigned to the same hybrid category, with almost identical assignment values.

### Assignment accuracy of simulated hybrids

**Fig. 3:**
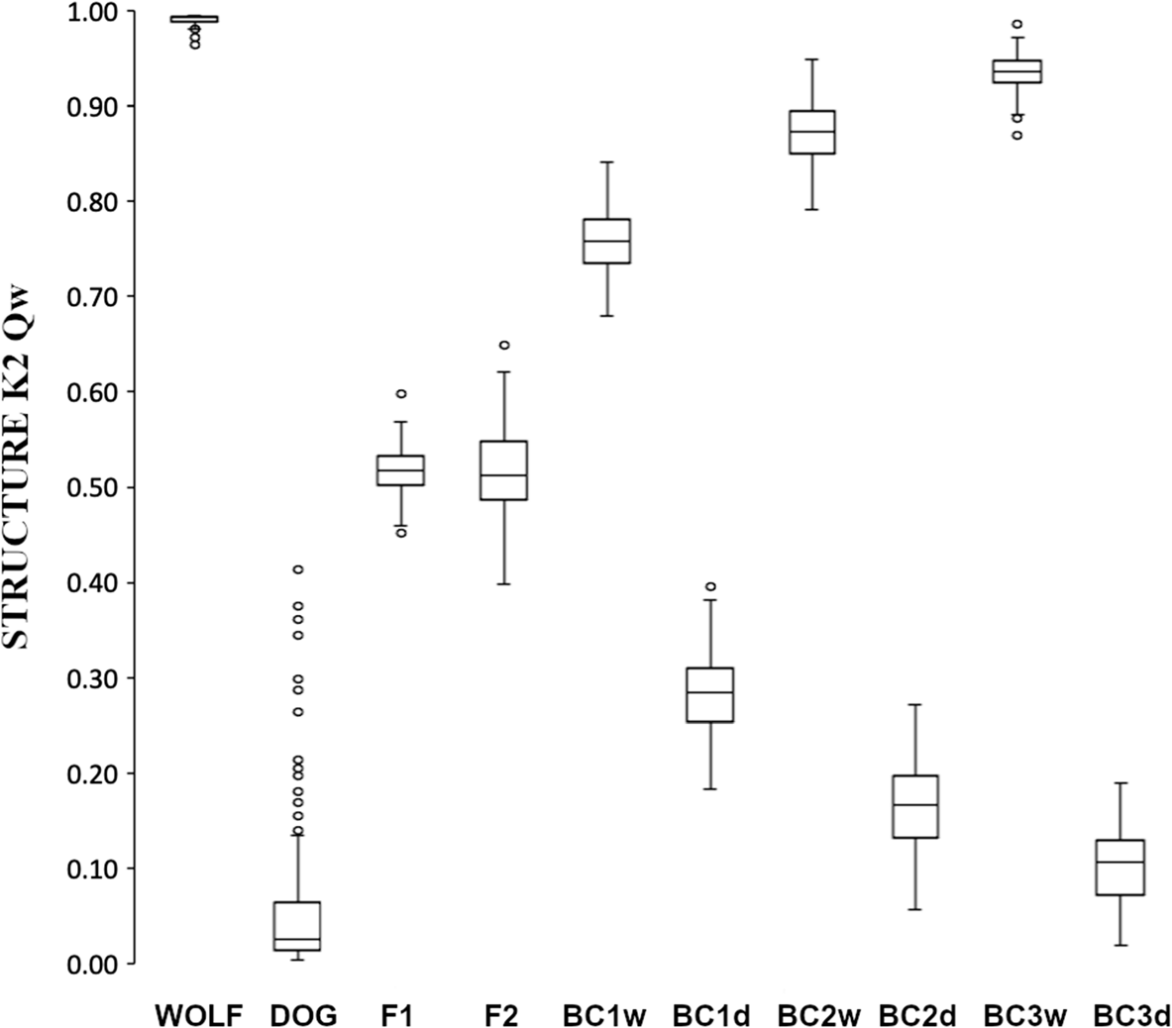
Individual assignment values to belong to the wolf cluster (*q*_w_) for wolves from Central and Eastern Europe (*n* = 162), dogs (*n* = 300) and simulated hybrids from each of the eight simulated genealogical classes (*n* = 100 per class) using STRUCTURE with *K* = 2. Means and quartiles are highlighted, while whiskers illustrate the range of values with outliers (circles)

When we analyzed simulated hybrids between wolves from Central and Eastern Europe and dogs, the STRUCTURE assignment distributions for wolves, simulated first-generation hybrids and first-generation backcrosses to wolves showed no overlap (F1 *q*_w_ = 0.46–0.60, BC1w *q*_w_ = 0.68–0.84, wolves *q*_w_ = 0.97–1.00) (Fig. 3), while there was some degree of overlap for later-generation hybrid classes (BC2w *q*_w_ = 0.79–0.95 and BC3w *q*_w_ = 0.87–0.99). When analyzed with NEWHYBRIDS, the number of correct assignments to the corresponding hybrid class was very high, even for third-generation backcrosses to wolf (89–92%) (Table 3). The highest accuracy in the correct assignment of wolf backcrosses was achieved using Jeffreys priors. The accuracy to distinguish a simulated hybrid from a pure individual by adding up the individual assignments of all hybrid categories was 100% for all wolf hybrid categories except BC3w (96–99%). Due to the larger variation found in dogs with these markers, the accuracy of categorizing dog backcrosses to the correct hybrid class dropped from 86 to 87% for BC1d to 76–77% for BC2d, and was zero for BC3d. The assignment accuracies were similar for wolves from Italy or the Iberian Peninsula (Table S10; Table S11).

**Table 3:** Assignment accuracy of simulated hybrid individuals between dogs and wolves from Central and Eastern Europe (Finland, Russia, Germany and Romania) from eight different hybrid classes to the correct category (> 0.5) or to any hybrid category (sum of assignments to hybrid categories > 0.7) based on results from NEWHYBRIDS runs with all the four possible prior combinations (see main text). Range of results from different runs is indicated

Hybrid Category	*n*	Correct Assignments (%) (*q*_i_ > 0.5)	Assigned to Hybrid Categories (%) (*q*_i_ > 0.7)
F1	100	100	100
F2	100	100	100
BC1w	100	99	100
BC2w	100	81–82	100
BC3w	100	89–92	96–99
BC1d	100	86–87	100
BC2d	100	76–77	79–80
BC3d	100	0	19

## Discussion

### Discriminating power of the selected SNP panel

We developed a 96 SNP panel from which 93 SNPs were finally selected based on performance (three SNPs were dropped as they had low genotyping success rate, < 0.7 across samples). The 93 selected SNPs allowed for reliable discrimination of wolves, dogs and their hybrids. This high discriminating power is due diverging allele frequencies in the wolf and dog groups, accompanied by the presence of private alleles for dogs. For all loci, alle frequencies were > 0.69 for one of the groups. While this panel was chosen to maximize the differentiation between wolves and dogs, significant differentiation between the wolf populations was detected. However, panels designed specifically to study population differentiation are available and better suited for this purpose (e.g. Illumina CanineHD chip, Affymetrix Canine SNP array or specifically designed SNP chips).

The fact that golden jackals and foxes had high amplification success and were not distinguishable from wolves requires caution. However, there are several genetic methods for differentiating these species from wolves that could be applied in routine laboratory analyses. Stronen et al. [28] have shown that only 11 microsatellite markers are sufficient to differentiate golden jackals from wolves. Even more convenient is to sequence a targeted region of mtDNA that allows to differentiate between the two species, e.g. cytochrome oxidase I (the barcoding gene), cytochrome b [29] or control region [30]. Amplifying the targeted mtDNA sequence a priori would not require much resources and could be implemented routinely for all non-invasive samples before SNP genotyping. As golden jackals were about as distinguishable from dogs as wolves were, this SNP panel could potentially also be used for detecting hybrids between these two species, albeit that would require further testing. Golden jackals have been shown to rarely hybridize with domestic dogs in the wild [30], which might be more common in the future, as golden jackals are expanding extensively throughout Europe [31], particularly if suitable mates are scarce, as seen for wolves [32]. Golden jackals and dogs have also been bred intentionally to develop a new breed (Sulimov dog) with good olfactory capabilities [33]: however, although used for narcotic detection at the Sheremetyevo Airport in Moscow, their superior olfactory skills have been questioned [34].

Although the discriminating power between wolves and dogs with this SNP panel was high (100% for F1 and F2, 99% for BC1w), and we were able to assign even third-generation backcrosses to wolves to the right category with high accuracy (89–92%), the assignment accuracy for second-generation backcrosses to wolves was slightly lower (81–82%). This hybrid category’s lower assignment accuracy is due to the fact that reliably distinguishing between second- and third-generation backcrosses is difficult; most of the incorrectly assigned hybrids from this category were assigned as third-generation hybrids (the remaining two or three individuals were assigned as first-generation hybrids). However, unless the criteria for defining a hybrid requires the distinction between these two hybrid categories, the lower assignment accuracy in this category is not relevant for management as the individuals would be anyway categorized as advanced hybrids. The software could not assign any third-generation backcrosses to dogs into the right category, possibly because the analysis was hampered by the large variation in allele frequencies in dogs. The amount of genetic variation is higher in dogs than in wolves when all dogs are combined, but variation within each single breed is less than that found in wolves [35]. In this SNP set, variation in dogs is emphasized by the fact that the SNPs are selected from the Illumina CanineHD Chip, the SNPs of which are in turn selected from the dog reference genome. Somewhat higher variation in dogs, wolves and different hybrid categories can be observed in a study testing 100 SNPs chosen from the Affymetrix Canine Mapping SNP Array 2.0, with SNPs also originally chosen from the dog reference genome [21]. Here we attempted to develop an efficient and reliable genotyping method that would allow to detect wild wolf-dog hybrids during routine wolf monitoring based on samples with low DNA quality and quantity. Therefore, reliable discrimination of dogs from backcrosses to dogs falls beyond the scope of this study.

### Extent of hybridization detected in the investigated wolf populations

During the 18th and 19th centuries, wolf populations in Central and Western Europe experienced large-scale contractions in their distribution and reductions in their population sizes. In the last decades, wolves have increased their distribution range and numbers in many parts of Europe [3]. During a recolonization phase when the population size is small, there is an increased risk for hybridization due to the lack of available mates [32]. The same holds for intensively hunted populations [13, 36]. Severe anthropogenic disturbance, such as intense hunting or poaching, has been shown to disrupt the normal social structure of wolf packs, turning them more tolerant towards individuals outside of the pack [37]. Because of these reasons, removing advanced generation backcrosses from nature needs to be carefully evaluated on case-by-case basis. Below, we discuss the evidence for hybridization detected in this study for each country. It should be noted that these samples are not representative of actual hybridization rates, as suspected hybrids were overrepresented for assay testing purposes.

#### Finland

Despite the fact that the Finnish wolf population experienced severe bottlenecks in the 1920s and 1970s [38, 39], we did not find any sign of admixture in museum wolves from the 1850s to 1980s. Wolves started to recolonize Finland in mid-1990 [40]. At present, the population size is estimated at 185–205 individuals [41]. Up to now, the only hybridization event reported in Fennoscandia was that of a lone female wolf breeding with a dog in southern Norway [42, 43]. Here, seven individuals were identified as hybrids; of those, three corresponded to a single hybridization event involving a male hybrid mating with a female wolf and their two backcross pups. Six of these individuals had already been identified as hybrids in Harmoinen et al. (in prep). That study included one additional hybridization event (comprising five pups), indicating that the total number of genetically confirmed hybridization events in Finland amounts to six, involving 12 individuals.

#### Romania

With a population size of around 2500 individuals [44], Romania has one of the largest wolf populations in Europe (note the northwest of the Iberian Peninsula encompasses comparable numbers of about 2200–2500 individuals [3];). In this study, we confirmed the hybrid status of a suspected individual (second-generation backcross to wolf), which was previously identified using microsatellites (A. Jarausch, unpublished No signs of introgression were obtained for the 22 remaining samples, suggesting that hybridization occurs, but may not be a widespread phenomenon. More samples collected across the entire region are needed to provide a reliable estimate of wolf-dog hybridization rate in this area.

#### Germany

After extirpation and absence for almost a century, wolves of Polish origin have been reported to reproduce in Germany since 2000 [45]. As of 2019, 105 packs were documented [46]. Only three cases of hybridization with male domestic dogs (Saxony in 2003 and Thuringia 2017/ 2019) have been documented in the frame of intense, microsatellite-based national genetic wolf monitoring [46]. No signs of recent dog introgression were found in the 100 German wolf samples analyzed in this study, confirming that hybridization rate in Central Europe is very low despite of the ongoing recolonization process.

#### Immigrants from the Alpine population

This study supports that the seven individuals sampled in Germany are immigrants from Italy or the Alpine region. These individuals have a mtDNA haplotype previously only seen in wolves from Italy, and had been previously assigned to the Italian wolf population based on microsatellite markers (unpublished). *F*_ST_ between Italian wolves and these seven individuals was very low and clustered together in the PCA analysis (Figure S1). These individuals probably originate from the Alpine region, which was recolonized by wolves in the 1990’s, after 70 years of absence [47]. There has been only one study showing low level of hybridization in the Alps ([12]; however, see hybrid detection in regions close by, [14, 19, 20]). In this study, the seven suspected immigrants were assigned to wolves when Italian wolves were used as a reference population. In the absence of wolves from Italy, these individuals were incorrectly assigned to later-generation backcrosses to wolves. Thus, even for marker sets with a low population signal, such as this SNP panel, including individuals belonging to the appropriate reference populations is of critical importance.

#### Iberian Peninsula

There is some evidence indicating that one individual may be an advance backcross to wolves (Fig. 2), while NEWHYBRIDS indicated this individual would be a wolf. A larger sample size would be needed for results to be conclusive.

#### Captive wolves

We found no sign of recent hybridization for the wolves from both Tierpark Berlin and Wildpark Poing.

### Suitability of the SNP panel for non-invasively collected samples

Our study confirms high SNP amplification success rates with good genotyping consistency for non-invasively collected samples with the Fluidigm microfluidic array technology, confirming similar findings in previous studies (e.g., [24, 25]), particularly when protocols are adapted for samples with low DNA quality and quantity [25, 48]. In the light of those studies, high levels of genotyping success indicate that more intensive replication effort is not necessary. This was further supported by the fact that identical or almost identical genotypes were obtained from invasive and non-invasive samples from the same individuals in this study (only one false allele and three missed alleles in three out of the 30 non-invasive samples examined). We note that, when genotyping success rate of the samples is high (Table 1), disagreements across replicates and thus errors are low (see [25] for an extensive discussion on this particular point). Notably, the museum samples (from 1850s and later) were genotyped with high call rate. Therefore, this method would be well suited in a variety of scientific studies, including those based on samples of lower DNA quality and/or quantity.

### Implications for wolf monitoring and research across Europe

Whereas the obtaining wolf-dog hybridization rates remains a central issue in wolf monitoring and management, relying on non-standardized microsatellite-based analysis of non-invasively collected samples has so far hampered the comparability of regional data, resulting in a lack of over-regional, European-wide hybridization rate estimates [2]. The application of this novel panel would solve the technical issues that prevent us from obtaining data that are comparable across regions.

We found an overall low population signal in this study. Nevertheless, our results show the importance of including samples from the relevant populations. Indeed, including reference samples from wolves from Central or Northern Europe, the Iberian Peninsula and Italy and/or the Alpine region when testing for admixture in these regions is of critical importance. In contrast to microsatellites, obtaining reference data can be easily achieved through extracting genotypes from already available genome-wide SNP or sequence data.

Laboratories that have already established the Fluidigm genotyping workflow could offer genotyping services to other institutions, or provide assistance in establishing those protocols. We assume that for most national wolf monitoring programs, only one or two 96 sample array runs per year would be sufficient to screen for potential hybridization events on a routine basis, which produces consumable costs of around 800 € (without tax) per array plus a couple of working days for one staff member [24, 25, 48].

Wolves and dogs have co-existed for millennia. Even if dog genomic introgression into wolves is more common than initially appreciated in studies using a small number of markers, our results show that wolves have kept their genetic distinctiveness, in agreement with genome-wide studies [19, 20, 23]. In addition to a correct management of dogs, maintaining viable population sizes of wolves and limiting human disturbance on wolf pack structure is probably the best way to minimize the risk of hybridization. Wolves play an important ecological role and perturbations to wolf social structure by removing individuals, particularly advanced backcrosses to wolves, could in some cases be detrimental and promote further hybridization. Plans to routinely monitor hybridization in Europe should be initiated to help identify areas where actions may be directed to better control feral dogs and to promote measures that would support ecological separation of dogs and wolves. Standardized, concerted assessment of hybridization rates across Europe may serve as a basis for further research aiming at understanding regional differences in hybridization rates and degrees of dog introgression in wolf populations.

## Conclusions

The designed 96 SNP panel is a highly discriminative new tool that could be used in routine wolf monitoring to detect wolf-dog hybrids up to third-generation backcrosses to wolves. We demonstrated a high genotyping success rate for all sample types, including different types of non-invasive samples commonly collected in monitoring practices and even museum samples, making the panel suitable for various types of studies. Moreover, the developed SNP panel is applicable at a European-wide scale, making it possible to produce comparable results of hybridization rates across the continent, as long as all the potential reference populations are included in the analyses. Extensive collection of wolf and dog reference samples is not required, as already published genotypes of wolves and dogs can be added to the analyses. The study reduces the gap between genomic research and real-world application by developing a fast and affordable method for wolf monitoring and management purposes.

## Methods

### SNP selection

Our initial SNP panel consisted of 300 wolf-dog ancestry-informative markers (AIMs) obtained from Harmoinen et al. (*in prep*). The SNPs were initially selected from a total of 173,662 SNPs on the CanineHD Whole-Genome BeadChip microarray (Illumina, Inc., San Diego, California, USA) which was used to genotype wolves sampled in most of their Eastern European range (Finland, Sweden, Russia, Estonia, Latvia, Poland, Belarus, Ukraine, Slovakia, Croatia, Bulgaria and Greece; *n* = 180) and dogs from 58 different breeds (collected in Finland, *n* = 352). In the study, unlinked (*r*^*2*^ < 0.2) data with MAF > 0.1 was used to select SNPs with the highest *F*_ST_ between wolves and dogs as AIMs (*F*_ST_ 0.67–0.86). Due to strict pruning, SNPs were evenly distributed across the 38 autosomal chromosomes. We then excluded SNPs located near another polymorphic site (minimum separation distance 100 base pairs; based on the dog genome, [49], with UCSC Genome Browser, [50]) to avoid problems in the interpretation of results and to simplify primer design (*n* = 63 excluded). This resulted in 237 markers, from which we selected 192 markers with the highest *F*_ST_ values for downstream testing using microfluidic arrays (Table S12).

### Assay development and testing

SNPtype™ genotyping assays were designed for the 192 selected AIMs and tested on microfluidic 96.96 Dynamic Arrays™ (Fluidigm Corp., South San Francisco, USA) following the recommendations and testing scheme in vonThaden et al. [25, 48]. The Fluidigm platform uses chips containing integrated fluidic circuits (IFCs), harbouring nanoscale PCR reaction chambers that allow the simultaneous genotyping of 96 samples and 96 loci [51]. We chose samples with high DNA concentration (*n* = 92, tissue and concentrated buccal swabs; ~ 20–80 ng/μl DNA) for the initial assessment of the 192 AIMs following in silico design. Samples included wolves (*n* = 51), non-pedigree dogs (*n* = 30), known hybrids (*n* = 7) and three species that may be a source of DNA contaminations in non-invasively collected samples (red fox, *Vulpes vulpes*, *n* = 1; golden jackals, *Canis aureus, n* = 2; and red deer, *Cervus elaphus, n* = 1; see next section for more information on the samples). All 192 AIMs were initially run without a multiplexed pre-amplification step to exclude primer interference as a cause of potential performance failure. Results were then examined to exclude markers that either: (i) produced ambiguous genotype clusters or fluorescence for non-template controls (*n* = 38); or (ii) showed genotype disagreements compared to the genotypes generated with the Illumina CanineHD chip (*n* = 6). Subsequently, the best performing 96 SNPs were selected and tested on the same reference sample set, but now including a multiplexed pre-amplification step (specific target amplification; STA) according to the manufacturer’s protocol, which is recommended for samples with moderate DNA concentration. In subsequent runs of samples with low DNA quality and quantity, we adjusted the manufacturer’s STA protocol as indicated in vonThaden et al. ([25]; i.e., 3.2 μl instead of 1.25 μl DNA template and 18 instead of 14 PCR cycles in the STA step).

### Application of the selected 96 SNP panel

Using the final 96 SNP panel, we genotyped samples collected both invasively (tissue) and non-invasively (scats, saliva from kills, urine, hairs) to generate sufficient data for subsequent analyses of marker performance and discriminative power (Table 4; Table S5). Tissue samples were selected from our collections of wolves, dogs and other canids, which were obtained from road-kills and other carcasses. For 11 individuals we had both invasively and non-invasively collected samples, which allowed to compare marker performance in samples with high versus low DNA quantity and quality, respectively. Wolf samples were collected from three areas within the European distribution range (Central European population: Germany, *n* = 117; Carpathian population: Romania, *n* = 28; and Karelian population: Finland, *n* = 65 and Russia, *n* = 5). We also included 9 samples collected in Germany previously assigned to the Italian wolf lineage (clustering analyses based on microsatellite genotypes, data not shown, and with the most frequent mitochondrial haplotype of the Italian lineage, haplotype HW22, see [52], corresponding to haplotype W14 described by [53]). These samples were obtained as part of the German official wolf monitoring program and we refer to them here as ‘immigrants from the Alpine population’. For samples collected in Finland, more than half (*n* = 34) were museum samples (tissue, teeth, bone, footpad, dry blood, skin and claw) collected between the 1850’s and 1980’s [54]. We also included wolf samples from two different zoos in Germany, Tierpark Berlin (*n* = 3) and Wildpark Poing (*n* = 1).

**Table 4:** Number of genotyped samples with (a) the 96-SNP panel and (b) the Illumina CanineHD BeadChip, as well as the number of individuals included in the analyses after removal of samples with low genotyping success and construction of consensus genotypes from repeatedly genotyped individuals. See Table S5 for a complete sample list

a) 96-SNP panel dataset			
Species	Sampling location	Genotyped samples (*n*)	Analyzed individuals (*n*)
Gray wolf	Germany	117	100
	Germany (immigrants from Alps/Italy)	9	7
	Romania	28	21
	Finland	65	61
	Russia	5	4
	Captive (Germany)	4	4
Dog	Germany	39	38
	Romania	2	2
Wolf-dog hybrid	Germany	4	3
	Romania	1	1
	Czech Republic	1	1
	Finland	7	7
Golden jackal	Germany	3	3
Fox	Germany	4	3
b) Illumina CanineHD BeadChip datasets			
Species	Sampling location (*n*)	Analyzed individuals (*n*)	
Gray wolf	Italy	70	
	Iberian Peninsula	25	
Dog	Finland	274	

As dog reference, we sampled non-pedigree dogs from Germany (*n* = 35) and Romania (*n* = 2), collected from animal shelters, private owners, and from a carcass found in the field. We also sampled four individuals belonging to wolfdog breeds (Saarloos Wolfdog, *n* = 2; and Czechoslovakian Wolfdog, *n* = 2).

Furthermore, we had 12 suspected wolf-dog hybrids, which were identified as such based on previously-conducted microsatellite genotyping (Germany, *n* = 3; Romania, *n* = 1; Czech Republic, *n* = 1; and Finland, *n* = 7). These individuals were found to have less than 0.85 posterior probability to be assigned to the wolf cluster when analyzed with Bayesian assignment procedures implemented in STRUCTURE (unpublished data, see Table S6 for more information on these individuals). Five of the suspected hybrids from Finland had also been genotyped with the Illumina CanineHD chip data and their hybrid status was supported (Harmoinen et al. *in prep*).

To test for cross-amplification of DNA from species that may be present in non-invasively collected wolf samples, we included samples (*n* = 20) from human (*Homo sapiens*), roe deer (*Capreolus capreolus*), red deer (*Cervus elaphus*), Eurasian goat (*Capra aegagrus hircus*), sheep (*Ovis sp.*), wild boar (*Sus scrofa*), red fox (*Vulpes vulpes*), golden jackal (*Canis aureus*) and other European carnivore species (Table S5).

Genomic DNA from tissue and blood samples was extracted using the DNeasy® Blood & Tissue Kit (Qiagen), from scat and urine samples using the DNA Stool Mini Kit (Qiagen), and from hairs and saliva swabs using the QIAamp DNA Investigator Kit (Qiagen). For the museum samples, DNA extraction procedures are described in Jansson et al. [54] and they were genotyped under the same conditions as non-invasive samples. All genotyping reactions were set up in a laminar flow hood that was previously irradiated with UV light for 40 min. The STA-PCRs were set up in a laboratory dedicated for non-invasive samples. PCRs were performed in a physically separated laboratory to avoid contaminations. To assess potential genotyping errors, 50 of the 149 tissue samples were genotyped 2–3 times, all scat samples were replicated 1–3 times and all the remaining non-invasive samples and museum samples 1–5 times. Some individuals were genotyped using several different sample types and consensus genotypes were constructed over all samples and replicates (see number of replicates per sample and samples per individual from Table S5). For that purpose we used a custom script following the simple rules that the same genotype (i) has to be observed at least twice, otherwise it is marked as missing data, and (ii) must be the most commonly observed genotype over all replicates.

### Assessment of assay performance

We removed three SNPs with low genotyping success rate (< 0.7; BICF2P1334457, BICF2S2305845 and BICF2G630504215) and thus performed all subsequent analyses using genotypes based on 93 SNPs. We also removed a few samples that failed to amplify in all reactions e.g. due to poor sample quality (see Table 1). Assay performance was assessed using three different measures:

(i) *Genotyping success rates for DNA from different sources* (tissue, concentrated buccal swab, saliva swab, hair, scat, urine, blood, museum samples). For each sample the proportion of scored loci over all loci was calculated, and an average was obtained for the corresponding tissue category (Table 1).
(ii) *Genotyping consistency*
  - *between non-invasive and tissue samples from the same individuals* (tissue samples *n* = 11, non-invasive samples *n* = 30). For 11 individuals, we compared the genotype from the tissue sample against each genotype from a non-invasively collected sample. We counted a false allele when an allele found in the genotype of a non-invasive sample was not present in the genotype of a tissue sample. A missing allele was counted in the cases in which two alleles were present in the tissue sample, and only one in the non-invasive sample. The proportion of false alleles was calculated as the number of false alleles divided by number of homozygous genotypes (*n* = 2664) and the proportion of missing alleles as the number of divided by the number of heterozygous genotypes (*n* = 104) in the tissue samples, due to the fact that the selected SNPs were biallelic. In addition, we counted the number of loci with missing data and divided by number of all loci to get the missing rate per sample. Proportion of loci with missing genotypes in the study was calculated by taking average over samples.
  - *between microfluidic array-based and Illumina CanineHD chip genotypes of the same individuals* (*n* = 22 tissue samples). Illumina CanineHD genotypes of the wolves were taken from Harmoinen et al. (in prep), extracting the genotypes for the corresponding 93 SNPs. To be able to calculate the genotyping error rates, we assumed that the genotype based on the Illumina chip was the true genotype.
(iii) *Cross-species amplifications*. We checked if any of the samples we included in the assays that were not wolves or dogs yielded genotypes.

Samples with < 0.8 genotyping success rate (proportion of scored loci per sample) were removed from all analyses (wolves, *n* = 14; potential wolf-dog hybrids, *n* = 1; potential cross-species contaminants, *n* = 15), except for two foxes which were included with genotyping rates of 0.77 and 0.78 (removed samples indicated in Table S5).

### Statistical analyses

For the statistical analysis of hybridization and population differentiation, we added additional genotypes to the dataset. The genotypes of 70 Italian and 25 Iberian wolves were extracted from the Illumina CanineHD chip data (Table 2; Table S5) and included in the analyses to test the performance of the SNP panel on wolves from Southern and Western Europe, which are genetically differentiated from other European wolf populations based on earlier genome-wide analyses [55]. Similarly, we extracted the genotypes of 274 dogs belonging to 55 breeds from the CanineHD chip dataset, in order to capture a larger proportion of the genetic diversity in dogs for the admixture and assignment analysis. Among the 274 dogs, there were ten individuals from two wolfdog breeds (Saarloos Wolfdog, *n* = 5 and Czechoslovakian Wolfdog, *n* = 5).

The total dataset used in the analyses consisted of 288 wild wolves, 4 wolves from zoos, 314 dogs (including 14 individuals from wolf-dog breeds), 12 suspected hybrids, 3 golden jackals and 3 foxes (Table 2).

We conducted principal component analysis (PCA) using the SMARTPCA package of the EIGENSOFT software [56] to visualize the genetic distance between individuals. Then we analyzed the dataset using a Bayesian clustering approach implemented in STRUCTURE ver 2.3.4 [57]. We conducted 5 independent runs for each value of K between 1 and 6 with a burn-in length of 50,000 and a run length of 500,000 Markov Chain Monte Carlo (MCMC) repetitions. We used the admixture model and correlated allele frequencies. We use the STRUCTURE HARVESTER program [58] and estimated the most likely number of populations (*K*) using the Evanno method [59]. The most likely number of clusters was two (Figure S5) and we used the mean over the 5 independent runs with *K* = 2 to estimate the assignment of each individual as wolf or dog. We also ran STRUCTURE analysis in the same way just for wolf genotypes in order to identify the most likely number of subpopulations among the wolves. As STRUCTURE is known to be affected by unequal sample sizes [60], we reduced the sample size in each geographical area to 20 individuals (the smallest sample size in our dataset) by excluding samples based on pairwise relatedness.

To test for population differentiation, and for differentiation between wolves and dogs, we calculated *F*_ST_ values between different groups of samples using ARLEQUIN 3.5.2.2 [61]. We considered all wolves as one group (*n* = 288) or as separate groups based on sampling location (for groups, see Table S4). In the case of dogs, we excluded individuals from wolf-dog breeds (*n* = 300). We performed 1000 bootstraps in order to get *p*-values around pairwise *F*_ST_ values.

We used the Bayesian model-based software NEWHYBRIDS without prior information about parental individuals (i.e., the z-option was not used), in order to see how the software categorized the empirical dataset into different hybrid classes. The software estimates the posterior probability of individuals falling into one of four default categories: two parental populations, F1, F2 and the two first-generation backcrosses to wolves (BCw) and dogs (BCd). We included four additional classes (second and third-generation backcrosses; BC2w, BC2d, BC3w and BC3d) using the corresponding derived frequencies. We analyzed all samples together but, because we found significant differentiation between different wolf populations in the analyses described above, we also conducted three additional analyses for wolf samples from (i) Central and Eastern Europe including Finland, Russia, Germany, Romania (*n* = 186); (ii) Italy (*n* = 70) and (iii) the Iberian Peninsula (*n* = 25). In all the analysis, we included all of the dog samples, including individuals from wolf-dog breeds (*n* = 314). In the analysis of wolf samples from Central and Eastern Europe (*n* = 186), we also included the suspected wolf-dog hybrids (*n* = 12), the wolves from the animal parks (*n* = 4) and the golden jackals and foxes (*n* = 6). In the analysis of wolf samples from Italy (*n* = 70), we also included the immigrants from the Alpine population sampled in Germany (*n* = 7). All the runs were conducted with four different prior combinations to explore the sensitivity of the results. We ran the program for an initial burn-in of 100,000 sweeps followed by 500,000 MCMC sweeps. A posterior probability value of ≥0.5 was used to assign individuals to a specific class.

To assess the power of the 93 SNPs in detecting recent hybrids between wolves and dogs, we used simulated genotypes generated with the software HYBRIDLAB v1.0 [62]. The simulated genotypes represented individuals of eight different hybrid classes (100 individuals for each class), as described above. We generated genotypes separately for wolves from Central and Eastern Europe (*n* = 162), Italy (*n* = 70) and the Iberian Peninsula (*n* = 25). For the parental population comprising wolves from Central and Eastern Europe we included tissue, hair and saliva samples (*n* = 162), and excluded scat samples to minimize the risk of potential DNA contamination in the field that may affect the allele frequencies. Independently of sample type, all wolf samples had > 0.97 assignment to the wolf cluster with STRUCTURE using *K* = 2, in analyses conducted separately for the different wolf datasets, Central and Eastern Europe, Italy, Iberian Peninsula. The other parental population comprised all the dog genotypes, except the ones from wolf-dog breeds (*n* = 300). Simulated hybrids were subsequently analyzed using STRUCTURE with *K* = 2, as well as NEWHYBRIDS. Simulated genotypes were run with the parental populations using the z-option, which allows to define wolf and dog parental individuals. Runs and analyses were performed in the same way as described above for the empirical data.

## Supplementary Information

**Additional file 1: Table S1.** Comparison of non-invasive samples to the consensus of corresponding invasive samples. Corresponding samples are on the same row. False allele: an allele seen in non-invasive sample but not in corresponding invasive sample; Missing allele: an allele seen in invasive sample but not detected in the corresponding non-invasive sample; Missing data: sample didn’t produce readable genotype, Hom: homozygous, Het: heterozygous.

**Additional file 2: Table S2.** Comparison of genotypes from same individuals genotyped with CanineHD chip (Illumina) and microfluidic array (Fluidigm). We assumed Illumina genotype as the true genotype of individual. False allele: an allele seen in Fluidigm genotype but not in corresponding Illumina genotype; Missing allele: an allele seen in Illumina genotype but not in Fluidigm genotype; Hom: homozygous; Het: heterozygous.

**Additional file 3: Table S3.** Genotyping success (proportion of successfully scored loci over all SNP loci) for samples from species that are potential sources of DNA in non-invasively collected samples.

**Additional file 4: Table S4.** *F*_ST_ values for dogs and wolves grouped based on the sampling location, except for Italian immigrants that were sampled in Germany. Analysis was performed also without Italian immigrants (*n* = 7) and Russian wolves (*n* = 4) due to low sample sizes in these groups. However, the F_ST_ values between the remaining groups were the same. When all wolves were combined as one group (*n* = 288), the overall *F*_ST_ to dogs (without wolf-dog breeds, *n* = 300) was 0.72 (*p* < 0.05). The overall *F*_ST_ between wolf-dog breeds (*n* = 14) and dogs (*n* = 300) was *F*_ST_ = 0.20, *p* < 0.05.

**Additional file 5: Table S5.** The first tab contains names, locations and sampling dates for the samples that were genotyped in this study. Column named “Replicates” in the first tab shows how many times the same sample was genotyped, and the number in the parenthesis shows how many times the same individual was genotyped. Samples that were not included in the analysis are indicated. The second tab contains names, locations and sampling dates for the samples from the wolves from Italy and the Iberian Peninsula that were genotyped with the CanineHD Whole-Genome BeadChip (Illumina). The third tab contains sample names and breeds for the dog samples that were genotyped with the Illumina CanineHD chip. In each tab, there are results from the STRUCTURE and NEWHYBRIDS runs, with all possible prior combinations, for all the individuals included in the runs. When the result differed between the runs, several results were included. If the analysis was been done using a consensus genotype based on several samples from the same individual, the same result is indicated for all samples.

**Additional file 6: Table S6.** Description of suspected hybrid samples and discussion of the corresponding results. Microsatellite results, used for comparison, are unpublished.

**Additional file 7: Table S7.** NEWHYBRIDS and STRUCTURE results for Finnish museum samples categorized in different time periods as in Jansson et al. [54].

**Additional file 8: Table S8.** NEWHYBRIDS and STRUCTURE results for wolves living in two animal parks in Germany.

**Additional file 9: Table S9.** NEWHYBRIDS and STRUCTURE results for other canid species that successfully amplified with the SNP panel.

**Additional file 10: Table S10.** Assignment accuracy for the selected 93 SNPs to categorize simulated individuals between dog and Italian wolves from 8 different hybrid classes to the correct category (> 0.5) or assign it to any hybrid category (sum of assignments to hybrid categories > 0.7) by the software NEWHYBRIDS. Analysis was run with the four possible prior combinations. Range of results from different runs is indicated.

**Additional file 11: Table S11.** Assignment accuracy for the selected 93 SNPs to categorize simulated individuals between dog and Iberian wolves for 8 different hybrid classes to the correct category (> 0.5) or assign it to any hybrid category (sum of assignments to hybrid categories > 0.7) by the software NEWHYBRIDS. Analysis was run with the four possible prior combinations. Range of results from different runs is indicated.

**Additional file 12: Table S12.** Description of SNPs and primer sequences used in this study. Allele frequencies for the 93 SNPs are also reported. The first tab contains 96 SNPs that were included in the final SNP panel. Three SNPs that were removed before the analysis are indicated. The remaining SNPs that were tested but not included in the final panel and their corresponding primer sequences are in the second tab.

**Additional file 13: Figure S1.** Principal component analysis (PCA) for wild wolves based on 93 SNPs selected to maximize discriminatory power between wolves and dogs. Wolves are labeled based on sampling locations, except immigrants from the Italian wolf population, which were sampled in Germany.

**Additional file 14: Figure S2.** Delta K values for 1 ≤ *K* ≤ 8 when analyzed wolves with STRUCTURE.

**Additional file 15: Figure S3.** STRUCTURE analysis for the wolf dataset using the best K value (*K* = 2).

**Additional file 16: Figure S4.** STRUCTURE analysis for the wolf dataset using *K* = 3.

**Additional file 17: Figure S5.** Delta K values for 1 ≤ *K* ≤ 8 when analyzed whole dataset with STRUCTURE. *K* = 2 had highest value.

## Data Availability

The dataset supporting the conclusions of this article is available in the Dryad repository: 10.5061/dryad.76hdr7stk.

## Abbreviations

AIM: Ancestry-informative marker
BCd: First-generation backcross to dog
BC2d: Second-generation backcross to dog
BC3d: Third-generation backcross to dog
BCw: First-generation backcross to wolf
BC2w: Second-generation backcross to wolf
BC3w: Third-generation backcross to wolf
F1: First generation hybrid between parental species
F2: Second generation hybrid (F1 x F1)
IFC: Integrated fluidic circuit
SNP: Single nucleotide polymorphism
STA: Specific target amplification
