# Supplementary figures and images for: Reliable wolf-dog hybrid detection in Europe using a reduced SNP panel developed for non-invasively collected samples
Source: BMC Genomics. 2021 Jun 25;22:473. doi: 10.1186/s12864-021-07761-5 (PMC8235813; doi:10.1186/s12864-021-07761-5)

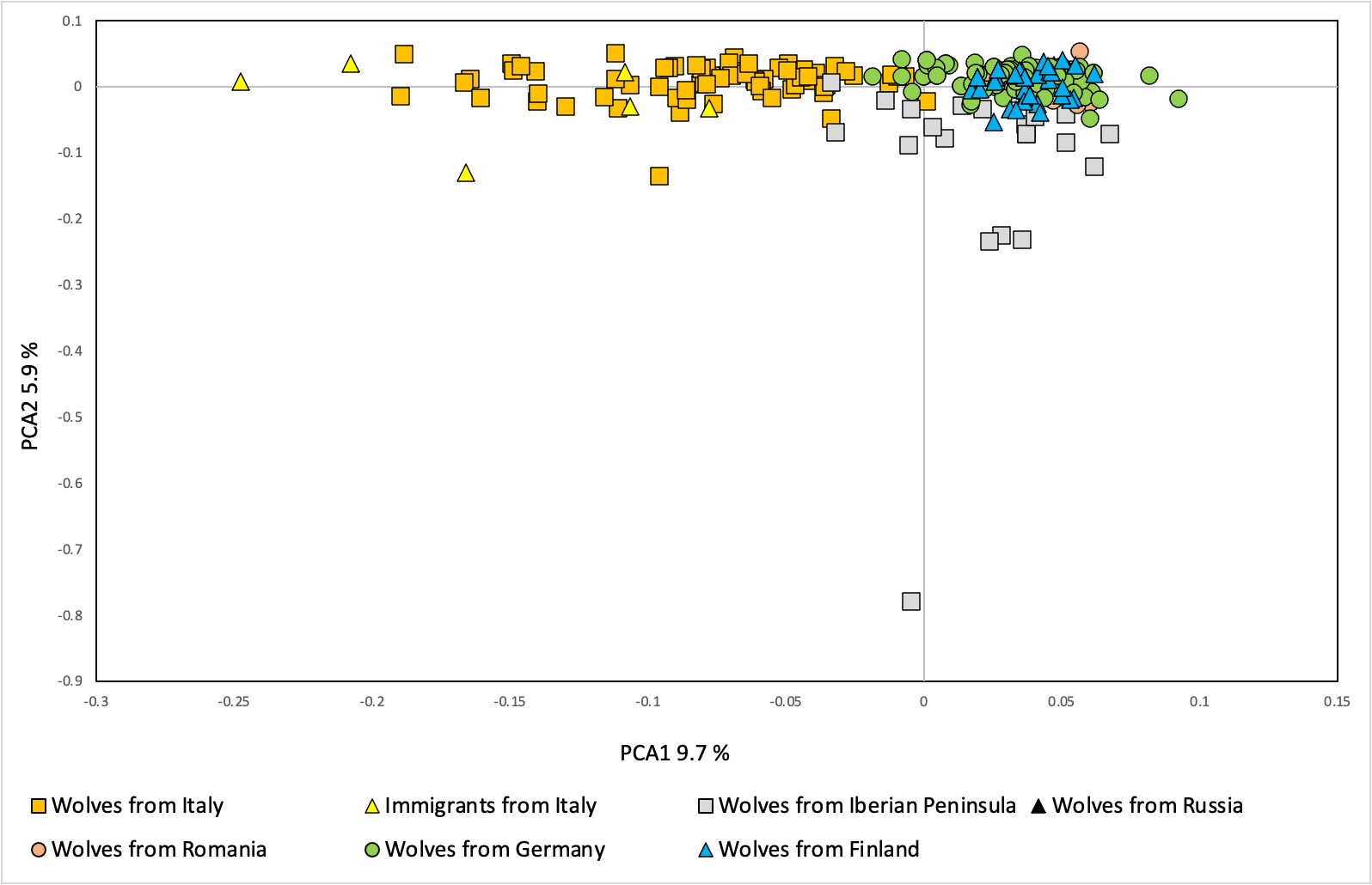

Supplement: Supplementary file 13 — Additional file 13: Figure S1. Principal component analysis (PCA) for wild wolves based on 93 SNPs selected to maximize discriminatory power between wolves and dogs. Wolves are labeled based on sampling locations, except immigrants from the Italian wolf population, which were sampled in Germany. [file 12864_2021_7761_MOESM13_ESM.tiff]

$$\text{DeltaK} = \text{mean}(|L''(K)|) / \text{sd}(L(K))$$

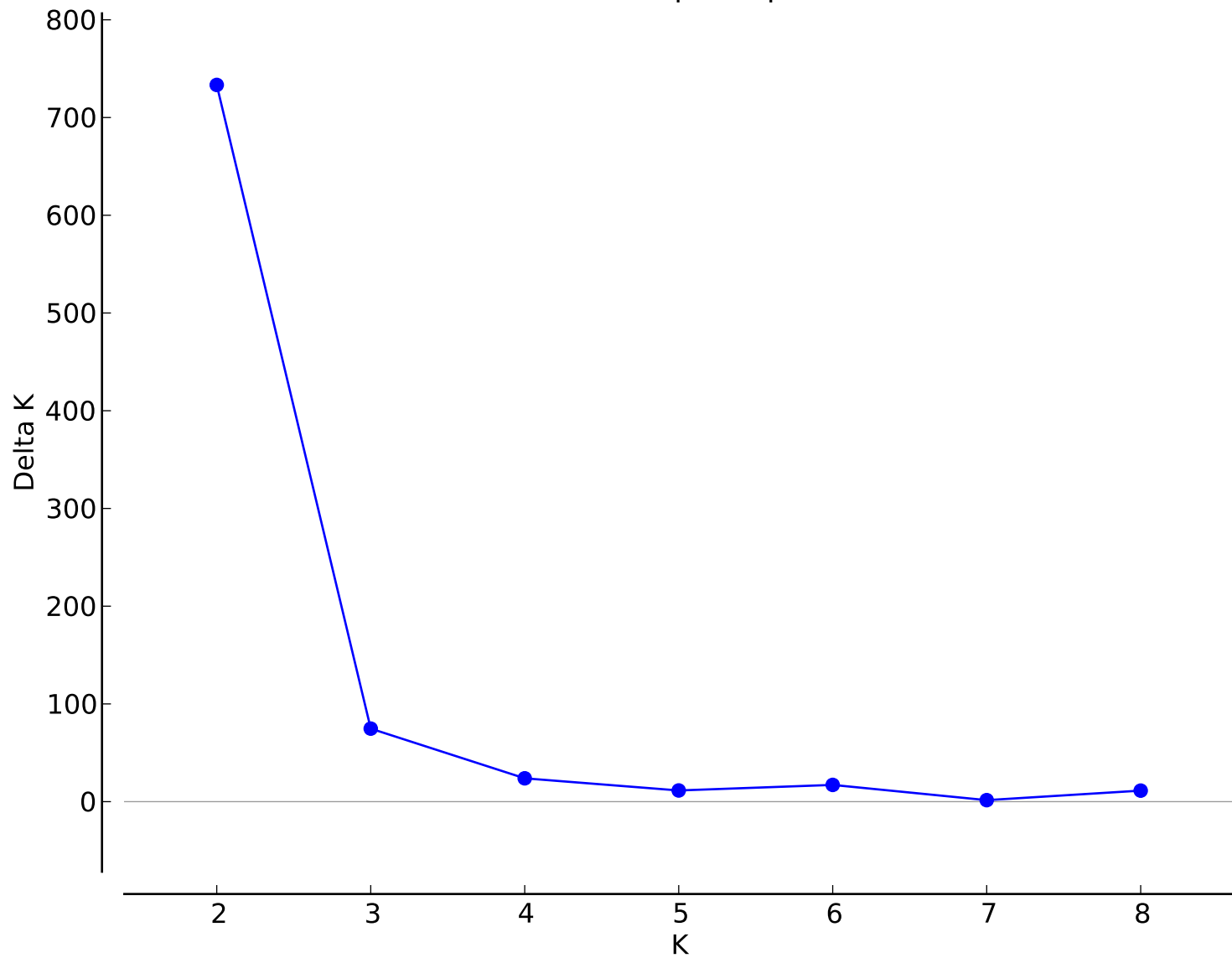

Supplement: Supplementary file 14 — Additional file 14: Figure S2. Delta K values for 1 ≤ K ≤ 8 when analyzed wolves with STRUCTURE. [file 12864_2021_7761_MOESM14_ESM.pdf]

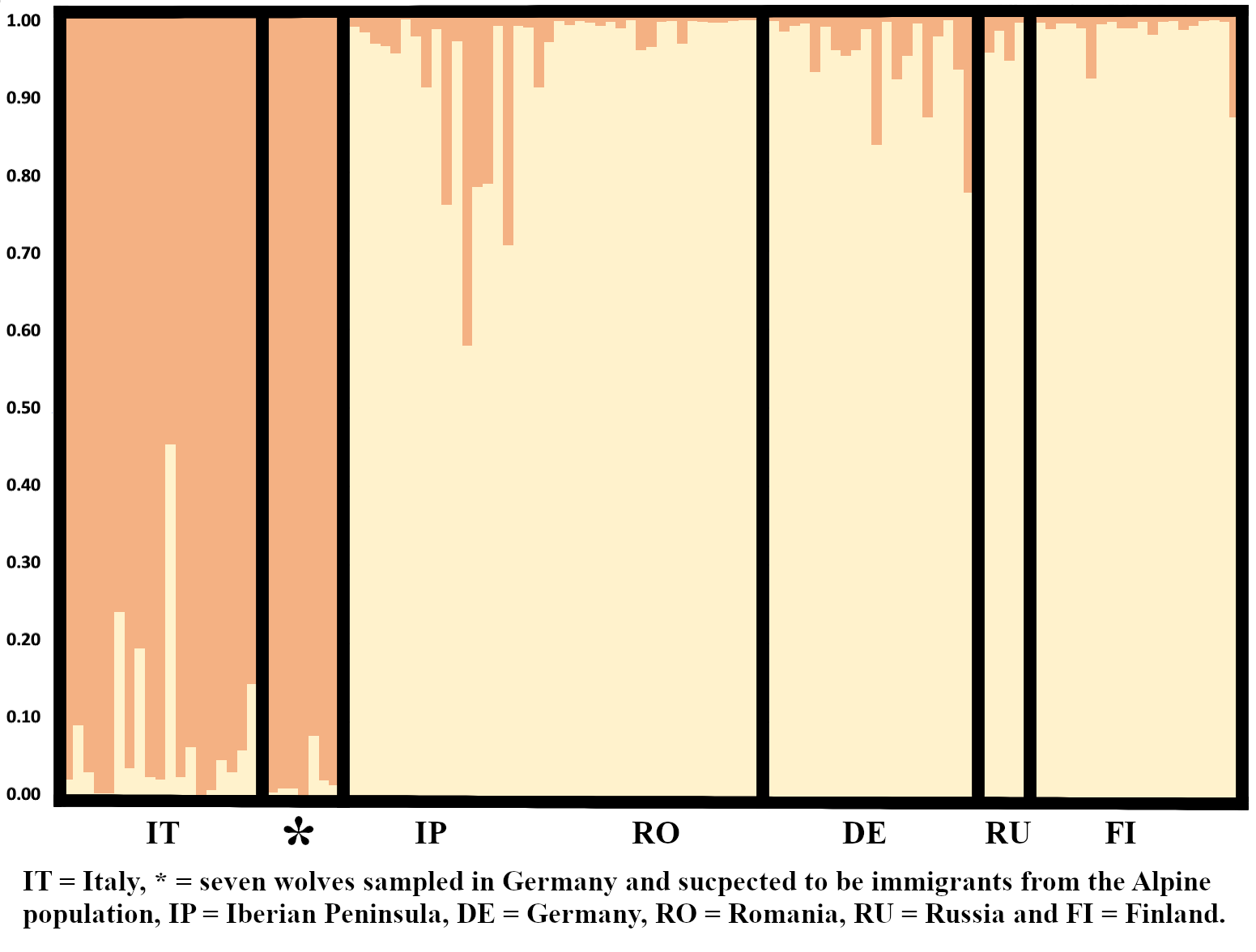

Supplement: Supplementary file 15 — Additional file 15: Figure S3. STRUCTURE analysis for the wolf dataset using the best K value (K = 2). [file 12864_2021_7761_MOESM15_ESM.tiff]

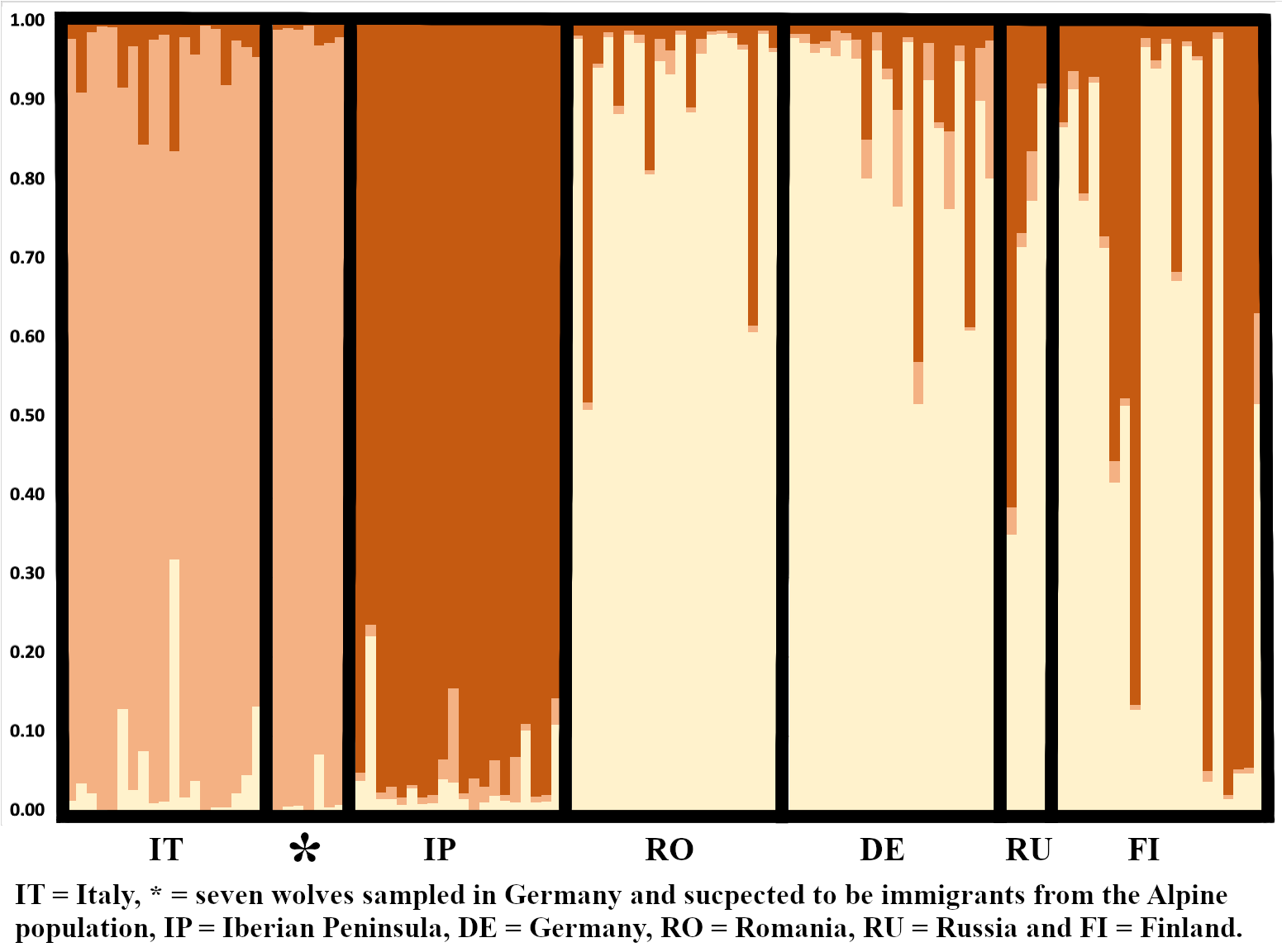

Supplement: Supplementary file 16 — Additional file 16: Figure S4. STRUCTURE analysis for the wolf dataset using K = 3. [file 12864_2021_7761_MOESM16_ESM.tiff]

$$\text{DeltaK} = \text{mean}(|L''(K)|) / \text{sd}(L(K))$$

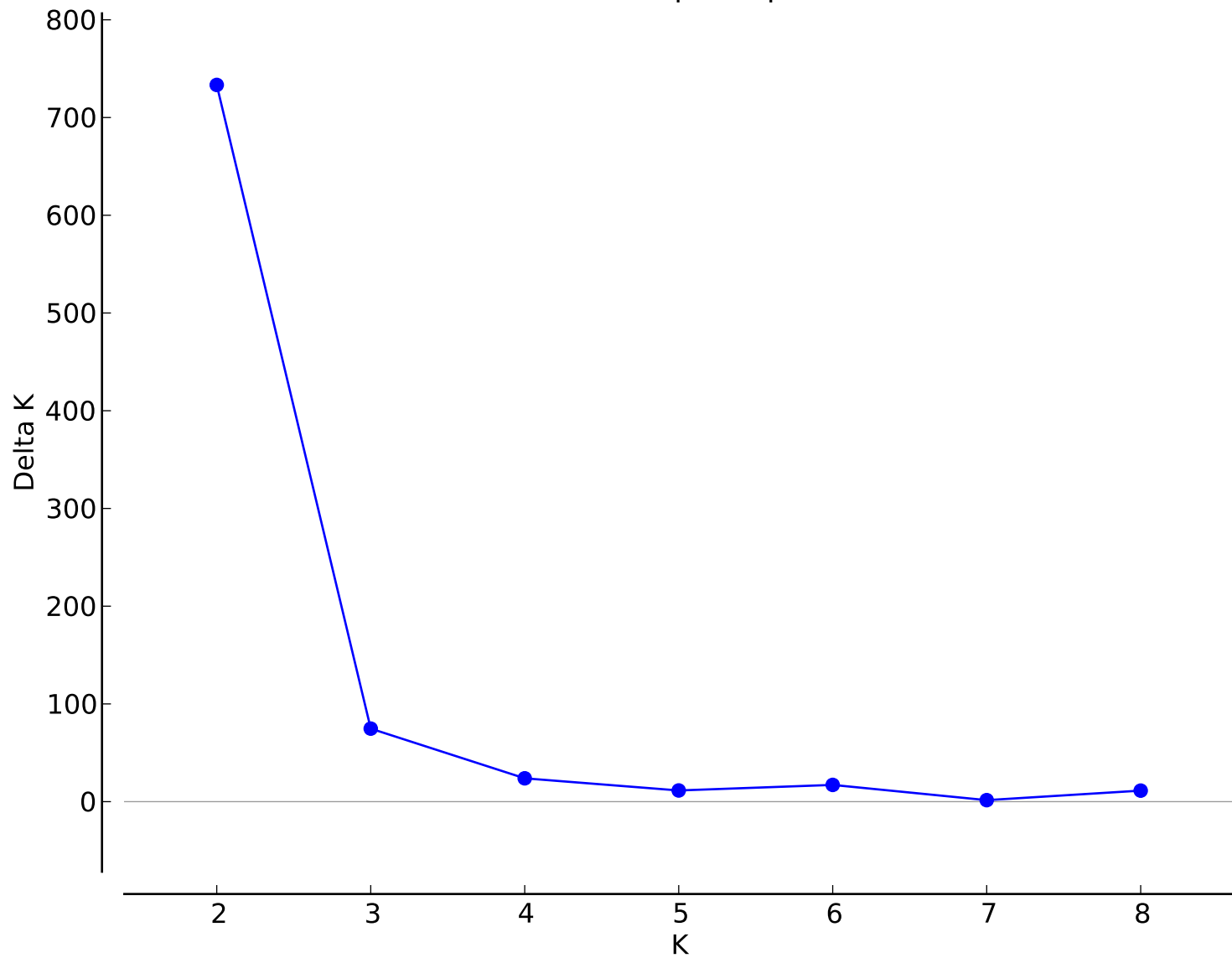

Supplement: Supplementary file 17 — Additional file 17: Figure S5. Delta K values for 1 ≤ K ≤ 8 when analyzed whole dataset with STRUCTURE. K = 2 had highest value. [file 12864_2021_7761_MOESM17_ESM.pdf]
